# Response and Remission Rates in Internet-Based Cognitive Behavior Therapy: An Individual Patient Data Meta-Analysis

**DOI:** 10.3389/fpsyt.2019.00749

**Published:** 2019-10-25

**Authors:** Gerhard Andersson, Per Carlbring, Alexander Rozental

**Affiliations:** ^1^Department of Behavioural Sciences and Learning, Linköping University, Linköping, Sweden; ^2^Department of Clinical Neuroscience, Karolinska Institutet, Stockholm, Sweden; ^3^Department of Psychology, Stockholm University, Stockholm, Sweden; ^4^Department of Psychology, University of Southern Denmark, Odense, Denmark; ^5^Institute of Child Health, University College London, London, United Kingdom

**Keywords:** response rates, recovery, predictors, individual patient data meta-analysis, internet-based cognitive behavior therapy

## Abstract

**Background:** Internet-delivered cognitive behavior therapy (ICBT) was developed over 20 years ago and has since undergone a number of controlled trials, as well as several systematic reviews and meta-analyses. However, the crucial question of response rates remains to be systematically investigated. The aim of this individual patient meta-analysis (IPDMA) was to use a large dataset of trials conducted in Sweden to determine reliable change and recovery rates across trials for a range of conditions.

**Methods:** We used previously collected and aggregated data from 2,866 patients in 29 Swedish clinical trials of ICBT for three categories of conditions: anxiety disorders, depression, and others. Raw scores at pre-treatment and post-treatment were used in an IPDMA to determine the rate of reliable change and recovery. Jacobson and Truax’s, (1991) reliable change index (RCI) was calculated for each primary outcome measure in the trials as well as the recovery rates for each patient, with the additional requirement of having improved substantially. We subsequently explored potential predictors using binomial logistic regression.

**Results:** In applying an RCI of *z* = 1.96, 1,162 (65.6%) of the patients receiving treatment were classified as achieving recovery, and 620 (35.0%) were classified as reaching remission. In terms of predictors, patients with higher symptom severity on the primary outcome measure at baseline [odds ratio (OR) = 1.36] and being female (OR = 2.22) increased the odds of responding to treatment. Having an anxiety disorder was found to decrease the response to treatment (OR = 0.51). Remission was predicted by diagnosis in the same direction (OR = 0.28), whereas symptom severity was inversely predictive of worse outcome (OR = 0.81). Conclusions: Response seems to occur among approximately half of all clients administered ICBT, whereas about a third reach remission. This indicates that the efficacy of ICBT is in line with that of CBT based in prior trials, with a possible caveat being the lower remission rates. Having more symptoms and being female might increase the chances of improvement, and a small negative effect of having anxiety disorder *versus* depression and other conditions may also exist. A limitation of the IPDMA was that only studies conducted in Sweden were included.

## Introduction

Internet-delivered cognitive behavior therapy (ICBT) has existed for more than 20 years (1), and treatment programs have been developed for a wide range of clinical and non-clinical conditions. Most forms of ICBT are administered in the form of guided self-help, with text and video presentations boosted by email support in a secure online platform resembling Internet banking (2). One way to describe ICBT is to pinpoint the similarities with online education, even if the treatment is largely based on self-help texts and cognitive behavioral therapy (CBT) manuals (3). Thus, ICBT programs tend to rely on psychoeducation and instructions for how to change thoughts, emotions, and behaviors in everyday life, and, like CBT, programs usually last 5 to 15 weeks with homework assignments and therapist feedback provided on a weekly basis (3).

Research suggests that therapist-supported ICBT—in contrast to unguided treatments (4)—can be as effective as face-to-face cognitive behavior therapy (5), yield long-term results (6), and work under clinically representative conditions (7). ICBT has also been tested for different target groups—for example, young persons (8), adults (9), older persons (10), and immigrants (11). Treatment programs have focused on specific problems, such as procrastination (12); diagnoses like post-traumatic stress disorder (PTSD) (13); or tailored according to a client’s specific problem profile (14). Another approach has been to deliver transdiagnostic treatments in which one treatment is used to target underlying common processes, such as avoidance (15). There are also studies on other psychotherapy forms, including psychodynamic psychotherapy (e.g., 16), interpersonal psychotherapy (17), versions of CBT such as acceptance and commitment therapies (18), and attention training (19). Finally, there are also programs based on physical exercise (20), mindfulness (21), and relaxation (22), even if the latter two are often incorporated into ICBT protocols (23). In addition to the controlled trials, several studies exist on moderators and mediators of outcome (e.g., 24), as well as some qualitative studies on the client’s experience during ICBT (25).

While it is common to report effects in clinical trials, there are also studies and reviews reporting negative effects and non-response to ICBT. In two previous individual patient data meta-analyses (IPDMA), we studied these two outcomes (26, 27). With regards to negative effects in the form of deterioration, 5.8% of treated research participants showed such effects (26), and non-response was present among 26.8% of participants (27). When completing these two reviews based on our dataset of controlled trials, a question emerged regarding response rates in our ICBT studies as this was not reported in the previous ones given uncertainties regarding definitions and scope of the two previous reviews. In contrast to the standard of reporting mean standardized differences with metrics like Cohen’s *d*, there is far less agreement on how to define response in psychological treatments studies in general, and specifically in CBT (28). Several questions emerge when defining what constitutes a “response” to treatment. In a review, 26, p. 73-74) mentioned several issues, such as (a) number of measures used to define response, (b) number of modalities (e.g., self-report *versus* observed behavior), (c) blinding of assessors, (d) degree of change from baseline, and (e) use of a clinical cut-off to determine if a client has reached a non-clinical state (sometimes referred to as high end-state or remission). In a seminal paper, Jacobson and Truax (29) outlined guidelines for defining change from baseline (reliable change index-RCI) and different ways to define what constitutes being within a non-clinical range or having reached high end-state function/remission. In the present review, we will use the term “remission” to refer to what can be counted as high end-state function, which allows us to be consistent with a previous IPDMA on depression by Karyotaki et al. (30).

In their IPDMA on guided ICBT for depression focusing on response and remission, Karyotaki et al. (30) included 24 RCTs (4,889 participants) and compared guided ICBT with a control group. The mean pooled response rate (based on RCI) at post-treatment was 56.19%, and the mean remission rate at post-treatment in the treatment groups (N = 26) was 38.51% based on the RCI criteria (1.96)—two standard deviations improvement from baseline for each measure. Given the dataset we coded based on our own trials, we decided to conduct a new IPDMA (31) knowing that we could use original data across studies to investigate reliable change and remission (high end-state function). As in our previous two IPDMAs (26, 27), we used data from 29 clinical trials. The final dataset with complete data consisted of 1,535 patients who had received ICBT, and trials were categorized into three groups: anxiety disorders, depression/mood disorders, and other conditions (i.e., erectile dysfunction, relationship problems, and gambling disorder). The aim of the current study was to determine the rates of treatment response and remission in clients who had received ICBT in clinical trials conducted by our group in Sweden. As a secondary exploratory aim, we examined potential predictors of response.

## Materials And Methods

### Individual Patient Data Meta-Analysis

As described in our two previous IPDMAs (26, 27), we used the scores for individual client and outcome variable across studies (32). In IPDMAs, it is study factors that might be predictive of treatment outcome using the raw data instead of group means, as has been done in previous IPDMAs—for example, on low-intensity psychological treatments (33) and Internet interventions for problem drinking (34). As in the previous IPDMAs, we aggregated available data from clinical trials that we conducted. A complete description surrounding our data collection procedure is presented in Rozental et al. (26). An obvious limitation of using this method is that we cannot assess the risk of bias, which is a common practice in systematic reviews (32). However, by including trials from our own group, we were able to obtain an overall view of response and remission, which we assume could be representative, particularly for Swedish settings. For a complete description of the inclusion and exclusion criteria used, see 26, p.163).

The raw scores from each client in the included trials were entered into one data matrix, and codes for background variables were aligned. Given the complexity of modeling reasons for missing data in such a heterogeneous group of research participants and the fact that we were focused on binary outcomes in this review, we decided to use a complete case approach instead of multiple imputation, as we were convinced that data were not missing completely at random (35). Moreover, complete case analysis has been recommended as the first approach when conducting meta-analyses, even if this approach is followed by sensitivity analyses to detect possible bias in estimates (36).

Sociodemographic variables were occasionally collapsed to facilitate comparisons and to obtain consistency across trials (see 26, 27). Trials were categorized into three categories: (1) anxiety disorders, (2) depression and mood disorders, and (3) other (i.e., erectile dysfunction, relationship problems, and gambling disorder). In **Table 1**, we present an overview of the clients’ sociodemographic variables in the trials included (the table overlaps with **Table 3** in 26, p. 169 but does not include the control groups). We present data from baseline in the trials and also the amount of missing data for the full sample.

**Table 1:** Sociodemographic characteristics of the participants included in the analysis (based on Table 3 in 26).

Baseline characteristic	ICBT (*n* = 1,864)	Full sample (*n* = 2,866)	Missing data for the full sample
Gender: *n* (% female)	1,120 (61.0)	1,800 (63.4) ^a^	27 (0.9) ^b^
Age (years): *M* (*SD*)	40.4 (16.0)	38.7 (12.8)	29 (1)
Civil status: *n* (%)			744 (27)
Single	419 (32.7)	668 (31.9)	
Relationship	861 (67.3)	1,424 (68.1)	
Children: *n* (% yes)	506 (54.4)	780 (55)	1,446 (50.5)
Cohabitant: *n* (% yes)	256 (65.6)	354 (12.4)	2,337 (81.5)
Highest educational level: *n* (%)			1,099 (38.3)
Elementary school	46 (4.1)	86 (4.9)	
High school/college	335 (30.1)	530 (30)	
University	720 (64.7)	1,137 (64.3)	
Postgraduate	12 (1.1)	14 (0.8)	
Employment: *n* (%)			1,968 (68.7)
Unemployed	65 (11.2)	93 (10.4)	
Student	78 (13.4)	138 (15.4)	
Employed	402 (69.1)	607 (67.6)	
Other	12 (2.1)	24 (2.7)	
Retired	25 (4.3)	36 (4)	
Primary diagnosis: *n* (%)			88 (3.1)
Anxiety disorders	930 (51.5)	1,681 (60.5)	
Generalized anxiety disorder	141 (7.8)	279 (10)	
Social anxiety disorder	541 (30.0)	965 (34.7)	
Anxiety disorder NOS	11 (0.6)	31 (1.1)	
Panic disorder (with/without agoraphobia)	61 (3.4)	116 (4.2)	
Posttraumatic stress disorder	32 (1.8)	64 (2.3)	
Anxiety disorder (with/without depression)	117 (6.5)	173 (6.2)	
Specific phobia	26 (1.4)	53 (1.9)	
Depression (with/without dysthymia)	439 (24.3)	544 (19.6)	
Other	436 (24.2)	553 (19.9)	
Erectile dysfunction	39 (2.2)	78 (2.8)	
Relationship problems	80 (4.4)	158 (5.7)	
Gambling disorder	317 (17.6)	317 (11.4)	
Sick leave: *n* (% yes)	41 (5.9)	67 (6.1)	1,768 (61.7)
Previous psychological treatment: *n* (% yes)	534 (55.8)	789 (54.7)	1,424 (49.7)
Previous or ongoing psychotropic medication: *n* (% yes)	336 (32.0)	522 (32.1)	1,239 (43.2)
Satisfaction with treatment: *M (SD)* ^c^	2.9 (1)	2.9 (1)	1,867 (88.2)^e^
Treatment credibility: *M (SD)* ^d^	7.2 (2.5)	7 (2.4)	1,535 (72.5) ^e^
Modules completed: *M (SD)* ^f^	6.6 (2.5)	6.5 (1.3)	1,194 (56.4) ^e^
Time per week: *M (SD)* ^g^	3.7 (3.0)	3.6 (3.1)	1,722 (81.3) ^e^

n.a., not applicable; NOS, not otherwise specified.

a Valid percent, i.e., percent of available data, excluding missing data.

b Percent, i.e., percent of complete dataset, including missing data.

c Self-rated 0–5.

d Self-rated 0–10.

e Based on patients receiving treatment.

f Weighted mean and standard deviation.

g Number of hours per week.

### Statistical Analysis

The RCI was chosen based on its widespread use for assessing reliable change (29, 37). As is common practice, the RCI was calculated by taking each individual change score and then dividing this score by the standard error of the difference, i.e., *SE*_diff_ = *SD*_1_√2√1-*r*. In the formula, *SD*_1_ corresponds to the standard deviation of a condition at pre-treatment, and *r* is the reliability estimate (38). We calculated RCIs for the primary outcome measure of each included trial and used the test–retest reliability for that specific trial measure (see **Table 2**; also reported in 27). Basically, the RCI sets the limit for when a change score is unlikely to be real (*p* = .05). Following the usual standards, we calculated RCIs for which a change equal to *z* = 1.96 on the basis of a standard deviation unit was used. Following this, each participant in the trials could be classified as either a responder or a non-responder to treatment, with the definition of response being specific to each study and measure used, a similar method to that used by Karyotaki et al. (30). Heterogeneity was tested by entering response rates into the program comprehensive meta-analysis (version 2.2.021; CMA).

**Table 2:** Test-retest reliabilities used for calculating improvement rates based on the reliable change index (same Table as in 27, p. 6).

Primary outcome	Test–retest reliability	Time period	Population	Reference
Beck Anxiety Inventory	*r* = 0.81	2 weeks	Normal	Saemundsson et al. (39)
Liebowitz Social Anxiety Scale—self-report	*r* = 0.93	8 weeks	Normal	Heeren et al. (40)
Panic Disorder Severity Scale—self-report	*ρ* = 0.94	2 days	Patient	Lee et al. (41). Reliability and validity of the self-report version of the Panic Disorder Severity Scale in Korea. *Depression and anxiety, 26(8)*, E120-E123.
Patient Health Questionnaire—nine items	*r* = 0.94	2 weeks	Patient	Zuithoff et al. (42)
International index of erectile functioning—five items ^a^	*r *= 0.84	4 weeks	Patient	Rosen et al. (43)
Beck Depression Inventory	*r* = 0.77	^b^	Normal	Beck and Steer (44)
Impact of Event Scale—revised	*r* = 0.89–0.94 ^c^ *M* = 0.92	6 months	Patient	Sundin and Horowitz, (45)
Generalized Anxiety Disorder—seven items	ICC = 0.83	1 week	Patient	Spitzer et al. (46)
Penn State Worry Questionnaire	*r* = 0.84	3 weeks	Normal	Pallesen et al. (47)
Body Sensations Questionnaire	*r* = 0.89	3 months	Patient	Arrindell (48)
Dyadic Adjustment Scale ^a^	*r* = 0.87	2 weeks	Patient	Carey et al. (49)
Snake Anxiety Questionnaire	*r* = 0.78	1 month	Normal	Klorman et al. (50)
Montgomery–Åsberg Depression Rating Scale—self-report	ICC = 0.78	1 week	Patient	Fantino and Moore, (51)
Spider Phobia Questionnaire	*r* = 0.94	3 weeks	Normal	Muris and Merckelbach, (52)
The NORC Diagnostic Screen for Gambling Problems	*r* = 0.98–0.99 ^d^ *M* = 0.98	1 week	Patient	Gerstein et al. (53)

NORC, a National Organization for Research at the University of Chicago.

a Reversed scales, higher scores indicate less problems.

b Information regarding the time period was unavailable.

c Separate estimates for the two subscales.

d Lifetime test statistic and past year test statistic.

We also followed the methods of Karyotaki et al. (30) when calculating remission, again using criteria set by Jacobson and Truax (29). Participants were classified as remitters if they moved two standard deviations below the mean of the clinical group to which they belonged in each study. The resulting cut-off scores indicates remission, which is a hard criterion of remission, often being equivalent to a symptom-free state. For six of the studies, it was not possible to use the two standard deviation criteria due to floor effects, so instead, we used one standard deviation as the criterion in these cases.

In order to investigate possible predictors, we applied binomial logistic regression and used either response or recovery rates as dependent variables. All variables were entered into the model simultaneously. In terms of the variables used, we selected a few clinically relevant demographic and clinical predictors (54) (31). We selected the same variables as in our previous IPDMAs on deterioration (26) and non-response (27). The predictors were (a) symptom severity at baseline, (b) civil status, (c) age, (d) sick leave, (e) previous psychological treatment, (f) previous or ongoing psychotropic medication, (g) educational level, (h) diagnosis, and (i) gender. We also added a separate analysis of the association between publication year and the two outcomes response and recovery.

We present odds ratios (OR) with 95% confidence intervals (CI) in order to reflect an increase or decrease in odds of response and remission in relation to a reference category. In the case of dichotomous predictors (such as gender), the OR reflects the odds of response or remission when a client is female *versus* male (reference). A positive OR thus means better response in women. For continuous predictors (symptom severity at baseline and age), the OR instead represents an increase of one standard deviation above their respective mean. The statistical analyses on predictors were performed using jamovi version 0.9.2.9 (55), with the proportions of response and remission performed on a complete case basis (see online **Supplementary Material**).

### Ethics

The data in the current IPMA were derived from several clinical trials, all of which had received ethical approval from the respective regional ethical review boards at each study location.

## Results

### Study Characteristics

The 29 clinical trials were coded according to the previously predefined inclusion and exclusion criteria and deemed eligible for the current IPDMA (see 26, 27 for further details). Raw scores from all clients were entered into one spread sheet. The exception was 46 clients who had received either psychodynamic psychotherapy or interpersonal psychotherapy *via* the Internet as a control condition. In total, 1,535 clients were included in this IPDMA. The following diagnoses were included (clinical trials, *k*): social anxiety disorder (9), depression (with/without dysthymia) (5), generalized anxiety disorder (3), anxiety disorder (with/without depression) (3), mixed anxiety disorders (e.g., panic disorder as well as social anxiety disorder) (2), specific phobia (2), posttraumatic stress disorder (1), panic disorder (with/without agoraphobia) (1), gambling disorder (1), erectile dysfunction (1), and relationship problems (1) (see 26, p.6). Briefly, most participants in the trials had been recruited from the general population based on self-referral (n = 27). A common practice in the trials was to use structured telephone interviews such as the Structured Clinical Interview for DSM-IV-Axis I Disorders (56) or the MINI-International Neuropsychiatric Interview (57). Some studies had used diagnosis-specific instruments such as the Clinician-Administered PTSD Scale (58). For a description of treatment content original studies, (see 26, 27). The amount of missing data for the primary outcome measures at post-treatment was 12.9%. A complete overview of the clinical trials is presented in **Table 3** (which to some extent overlaps with **Table 3** in 27 but with different results presented).

**Table 3:** Characteristics and rates of improvement and recovery for the clinical trials included in the individual patient data meta-analysis.

Study	Recruitment	Screening interview	Primary diagnosis	Treatment (*n*)	Control (*n*)	Modules/weeks or sessions	Primary outcome	Additional outcomes	Improvement *n* (%)*	Recovery *n* (%)*
IMÅ (21)	General population	SCID-I	Panic disorder, social anxiety disorder, generalized anxiety disorder, anxiety disorder NOS	Unguided mindfulness with FAQ (42)	Wait-list with discussion forum (46)	8 modules/8 weeks	BAI	BDI, QOLI, ISI	26 (66.7%)	23 (59.0%)
ACT Smart (59)	General population	SCID-I	Panic disorder, social anxiety disorder	Unguided ACT (48), guided ACT (48)	Wait-list (47)	8 modules/10 weeks	LSAS-SR, PDSS-SR ^a^	PHQ-9, GAD-7, QOLI	38 (49.4%)	8 (10.4%)
ACTUA (60)	General population	SCID-I	Depression	Guided physical activity (164), guided behavioral activation (158) ^b^	n.a.	8 modules/12 weeks	PHQ-9	GAD-7, QOLI ^c^	146 (75.3%)	84 (43.3%)
ADAM (61)	General population	Semi-structured interview, IIEF-5	Erectile dysfunction	Guided CBT (39)	Wait-list with discussion forum (39)	7 modules/7 weeks	IIEF-5	IIEF, RAS, BDI, BAI	4 (12.1%)	0 (0.0%)
Challenger (62)	General population	MINI	Social anxiety disorder	Unguided CBT with smartphone application (68), unguided bibliotherapy (70)	Wait-list (69)	9 modules/6 weeks	LSAS-SR	GAD-7, PHQ-9, QOLI, BBQ, Mini-SPIN	40 (62.5%)	18 (28.1%)
Stella (63)	General population	SCID-I and II	Depression	Guided CBT (33), group therapy (36), guided CBT as preferred choice (16)	n.a.	8 modules/8 weeks, 8 sessions	BDI	MADRS-S, BAI, HAM-D, QOLI,	27 (57.5%)	0 (0.0%)
Depressionshjälpen (64)	General population	SCID-I	Depression	Guided CBT (40)	Wait-list (40)	7 modules/8 weeks	BDI	MADRS-S, BAI, QOLI, WAI	26 (66.7%)	2 (5.1%)
Tellus (65)	General population	CAPS	Posttraumatic stress disorder	Guided CBT (31)	Wait-list with support (31) ^d^	8 modules/8 weeks	IES-R	PDS, BDI, BAI, QOLI	21 (75%)	11 (39.3%)
Klara (66)	General population	SCID-I	Depression	Guided CBT (29), guided CBT* via *email (30)	Wait-list (29)	7 modules/8 weeks	BDI	MADRS-S, BAI, QOLI	47 (83.9%)	4 (7.1%)
Oroshjälpen (18)	General population	SCID-I	Generalized anxiety disorder	Guided ACT (52)	Wait-list (51)	7 modules/9 weeks	GAD-7	PSWQ, GAD-Q-IV, BAI, MADRS-S, PHQ-9, QOLI	27 (64.3%)	15 (35.7%)
Nova 1 (67)	General population	SCID-I	Anxiety disorder with/without comorbid depression	Guided CBT (53)	n.a.	10 modules/10 weeks ^e^	BAI	MADRS-S, CORE-OM, QOLI	21 (42.0%)	14 (28.0%)
Nova 2 (68)	Primary care	SCID-I	Anxiety disorder with/without comorbid depression	Guided CBT (51)	Wait-list (50)	10 modules/10 weeks ^f^	BAI	MADRS-S, CORE-OM, QOLI	15 (39.5%)	1 (2.6%)
Origo 1 (23)	General population	SCID-I	Generalized anxiety disorder	Guided CBT (44)	Wait-list (45)	8 modules/8 weeks	PSWQ	GAD-Q-IV, STAI, BAI, BDI, MADRS-S, QOLI	23 (62.2%)	14 (37.8%)
Origo 2 (69)	General population	SCID-I	Generalized anxiety disorder	Guided CBT (27) ^g^	Wait-list (27)	8 modules/8 weeks	PSWQ	GADQ-IV, MADRS-S, BDI, BAI, STAI, QOLI	11 (47.8%)	4 (17.4%)
Panik 2 (70)	General population	SCID-I, CIDI	Panic disorder	Guided CBT (25), face-to-face CBT (24)	n.a.	10 modules/10 weeks, 10 sessions	BSQ	ACQ, MI, BAI, BDI, QOLI	17 (68.0%)	10 (40.0%)
Pia^1^	General population	SCID-I	Relationship problems	Guided CBT (80)	Wait-list with discussion forum (78)	10 modules/10 weeks	DAS	MSI, BDI, BAI, QOLI	21 (31.3%)	7 (10.5%)
Progredi (20)	General population	SCID-I	Depression	Guided CBT with physical activity (24)	Wait-list (24)	9 modules/9 weeks	MADRS-S	BDI, BAI, QOLI ^c^	15 (62.5%)	10 (41.7%)
Fobal snake (71)	General population	SCID-I	Specific phobia	Guided CBT (13), face-to-face CBT (13)	n.a.	4 modules/4 weeks, 2 sessions ^h^	SNAQ	ADIS, FSS, BAI, BDI	10 (83.3%)	7 (58.3%)
Sofie 13 (72)	General population	SCID-I	Social anxiety disorder	Guided CBT with CBM (61), Guided CBT without CBM (65),	n.a.	9 modules/9 weeks ^i^	LSAS-SR	SIAS, SPS, MADRS-S, QOLI	88 (73.5%)	41 (34.5%)
Sofie 9 (73)	General population	SCID-I and II	Social anxiety disorder	Guided CBT (40)	Attention bias modification (39)	9 modules/9 weeks	LSAS-SR	SIAS, SPS, SPSQ, BAI, MADRS-S, QOLI	27 (73.0%)	0 (0.0%)
Sofie 12 (74)	General population	SCID-I, MINI	Social anxiety disorder	Guided CBT with smartphone application (24) ^j^	n.a.	9 modules/9 weeks	LSAS-SR	k	19 (79.2%)	7 (29.2%)
Sofie 1 (75)	General population	SCID-I	Social anxiety disorder	Guided CBT (32)	Wait-list (32)	9 modules/9 weeks	LSAS-SR	SIAS, SPS, SPSQ, BAI, MADRS-S, QOLI	22 (75.9%)	7 (24.1%)
Sofie 2 (76)	General population	SCID-I	Social anxiety disorder	Guided CBT (29)	Wait-list (28)	9 modules/9 weeks	LSAS-SR	SIAS, SPS, SPSQ, BAI, MADRS-S, QOLI,	19 (65.5%)	7 (24.1%)
Sofie 3 (77)	Students	SCID-I	Social anxiety disorder	Guided CBT (19), guided CBT with group sessions (18)	n.a.	9 modules/9 weeks, 5 sessions	LSAS-SR	SIAS, SPS, SPSQ, BAI, MADR-S, QOLI	22 (59.5%)	3 (8.1%)
Sofie 4 (78)	General population	SCID-I and II	Social anxiety disorder	Guided CBT with discussion forum (40), unguided bibliotherapy (40)	Wait-list (40)	9 modules/9 weeks	LSAS-SR	SIAS, SPS, SPSQ, BAI, MADR-S, QOLI	22 (55.0%)	10 (25.0%)
Sofie 5 (78)	General population	SCID I	Social anxiety disorder	Guided AR (29), guided CBT with discussion forum (29), unguided bibliotherapy (29), unguided bibliotherapy with discussion forum (28)	n.a.	9 modules/9 weeks	LSAS-SR	SIAS, SPS, SPSQ, BAI, MADR-S, QOLI	34 (63.0%)	27 (50.0%)
Elsa (79)	General population	SCID-I	Anxiety disorder with/without comorbid depression	Guided CBT (33)	Wait-list with support (33)	8 modules/8 weeks ^l^	BAI	MADRS-S, CORE-OM, PHQ-9, GAD-7, QOLI	6 (27.3%)	3 (13.6%)
Fobal spider (80)	General population	SCID-I	Specific phobia	Guided CBT (13), face-to-face CBT (14)	n.a.	5 modules/4 weeks, 2 sessions ^h^	SPQ	ADIS, FSS, BAI, BDI	12 (92.3%)	9 (69.2%)
Gambling (81)	General population	n.a.	Gambling disorder	Guided CBT with discussion forum (317)	n.a.	8 modules/8 weeks	NODS	HADS, QOLI	221 (93.6%)	194 (82.2%)
Total									1,027 (69.9%)	540 (35.2%)

SCID-I, structural clinical interview for DSM-IV axis I disorders; NOS, not otherwise specified; FAQ, frequently asked questions; BAI, beck anxiety inventory; BDI, beck depression inventory; QOLI, quality of life inventory; ISI, insomnia severity index; n.a., not applicable; ACT, acceptance and commitment herapy; LSAS-SR, Liebowitz social anxiety scale—self-report; PDSS-SR, panic disorder severity scale—self-report; PHQ-9, patient health questionnaire—nine items; GAD-7, generalized anxiety disorder—seven items; BA, behavioral activation; MADRS-S, montgomery–åsberg depression rating scale—self-report; IIEF-5, International Index of erectile functioning—five items; CBT, cognitive behavior therapy; IIEF, International index of erectile functioning; RAS, Relationship assessment scale; MINI, the MINI-International neuropsychiatric interview; BBQ, Brunnsviken brief quality of life inventory; Mini-SPIN, mini-social phobia inventory; HAM-D, Hamilton rating scale for depression; WAI, Working alliance inventory; CAPS, Clinician-administered PTSD scale for DSM-IV; IES-R, Impact of event scale—revised; PDS, Posttraumatic diagnostic scale; PSWQ, Penn ptate worry questionnaire; GAD-Q-IV, Generalized anxiety disorder questionnaire; CORE-OM, Clinical outcome in routine evaluation—outcome measure; STAI, State-trait anxiety inventory; CIDI, Composite international diagnostic interview; ACQ, Agoraphobic cognitions questionnaire; BSQ, Body sensations questionnaire; MI, Mobility inventory; DAS, Dyadic adjustment scale; MSI, Marital status inventory; SNAQ, Snake anxiety questionnaire; ADIS, anxiety disorders interview schedule; FSS, Fear Survey schedule; CBM, Cognitive bias modification; SIAS, Social interaction anxiety scale; SPS, Social phobia scale; SPSQ, Social Phobia screening questionnaire; AR, applied relaxation; SPQ, Spider phobia questionnaire; NODS, the NORC diagnostic screen for gambling problems.

*Based on complete case analysis, i.e., only complete data.

a Separate analyses of deterioration were conducted for the two primary outcome measures depending on the diagnosis of the patient.

b Four treatment conditions were included in the study, with/without treatment rationale, respectively, but are pooled in the current analysis.

c An additional outcome measure, International Physical Activity Questionnaire, IPAQ, was used in the study but is not included in the current analysis.

d Passive control with the possibility to contact the research team if needed.

e Patients were able to choose 10 out of 16 modules to be completed during 10 weeks.

f Patients were able to choose 10 out of 19 modules to be completed during 10 weeks.

g An additional treatment group, guided psychodynamic therapy, was also used in the study but is not included in the current analysis.

h One brief orientation session and one session of 3-h prolonged exposure.

i In addition to 2 weeks of CBM.

j An additional treatment group, interpersonal psychotherapy, was also used in the study but is not included in the current analysis.

k Additional outcome measures were included in the original study but lost in the raw data file.

l Patients were able to complete up to eight modules selected by the therapist.

^1^Andersson G, Burman M, Norlander A-K, Magnusson K, Stalby M, Svensk M, et al. Internet-delivered couples therapy: a randomized controlled trial. (2019). Unpublished manuscript.

### Response and Remission Rates

Using the RCI criteria for detecting response, 1,027 (69.9%; 95% CI: 67.61–72.19) of the 1,535 participants receiving treatment were categorized as treatment responders when using an RCI of *z* = 1.96. The lowest rates were found in the trials on erectile dysfunction (12.1%) and older adults with anxiety (27.3%), whereas the highest rates were found in the trials on gambling (93.6%) and spider phobia (92.3%). As seen in **Table 3**, the proportions varied, which was confirmed by the CMA program showing a significant heterogeneity (*I* *^2 =^* 87.5%; Q = 223, *p* < .001).

Remission was achieved in 540 participants (35.2%; 95% CI: 32.81–37.59) when adjusting for floor effects (the unadjusted proportion was 31.9%). There was a large variation with ranging from 0% (erectile dysfunction, depression, and bias modification for social anxiety disorder) to 82% (gambling) and 69% (spider phobia). As with the response rates, a significant heterogeneity was found (*I* *^2 =^* 91.6%; Q = 334, *p* < .001).

### Predictors of Response

Binomial logistic regressions were calculated with the predefined variables entered as predictors of response. Results are presented in **Table 4** with OR and 95% CI for each predictor. The results that indicate a higher symptom severity on the primary outcome measure at baseline was predictive of better outcome. The odds for responding to treatment decreased when having an anxiety disorder as compared to depression/mood disorder and other diagnoses (i.e., erectile dysfunction, relationship problems, and gambling disorder), and the odds increased if the subject was female. The other variables were not predictive of response.

**Table 4:** Significance levels, odds ratios, and 95% confidence intervals (CI) for predictors of response.

Predictor (*reference*)	OR	Lower CI	Upper CI	*p*
Symptom severity at baseline (*lower severity*)	1.36	1.12	1.65	<.01
Civil status (*single*)	1.20	0.75	1.91	.45
Previous psychological treatment (*no*)	0.77	0.50	1.19	.23
Previous or ongoing psychotropic medication (*no*)	0.94	0.58	1.54	.82
Sick leave (*no*)	0.41	0.16	1.06	.07
Educational level (*below university level*)	0.72	0.47	1.11	.14
Age (*lower age*)	1.01	0.99	1.02	.28
Diagnosis, anxiety disorders (*depression/mood disorder and other*)	0.51	0.33	0.79	<.01
Gender (*male*)	2.22	1.43	3.44	<.001

OR, odds ratio; CI, 95% confidence interval; p, p-value.

We also repeated the analyses with remission as outcome (see **Table 5**). In this analysis, symptom severity at pre-treatment was marginally associated with less remission (OR = 0.81). As with the analysis for response, the odds for remission were lower if the subject suffered from anxiety. Gender and the other variables did not reach statistical significance. Publication year was unrelated to response and remission.

**Table 5:** Significance levels, odds ratios, and 95% confidence intervals (CI) for predictors of recovery.

Predictor (*reference*)	OR	Lower CI	Upper CI	*p*
Symptom severity at baseline (*lower severity*)	0.81	0.66	1.00	.05
Civil status (*single*)	0.98	0.59	1.64	.94
Previous psychological treatment (*no*)	0.65	0.41	1.04	.07
Previous or ongoing psychotropic medication (*no*)	1.33	0.77	2.30	.30
Sick leave (*no*)	0.65	0.21	2.00	.44
Educational level (*below university level*)	0.73	0.46	1.16	.18
Age (*lower age*)	1.00	0.99	1.02	.98
Diagnosis, anxiety disorders (*depression/mood disorder and other*)	0.28	0.16	0.47	< .001
Gender (*male*)	1.38	0.83	2.28	.21

OR, odds ratio; CI, 95% confidence interval; p, p-value.

## Discussion

The aim of this IPDMA was to obtain estimates of response and remission rates for ICBT with a range of conditions categorized into three groups (anxiety, depression, and other). In line with a previous IPDMA on depression by Karyotaki et al. (30) which included trials from different countries, we found that 65.6% of the treated research participants could be classified as treatment responders. This is slightly higher than the 56.19% reported by Karyotaki et al. (30). While Karyotaki et al. (30) imputed missing data, they also reported that the estimates between complete case analysis and the imputed dataset were minor. In addition, we found that 35.0% of participants could be classified as having remitted, which is slightly lower than the 38.51% remission rate reported by Karyotaki et al. (30). Moreover, we had a problem with floor effects and if not considering that our estimate is even lower (31.9%). Overall, the results are rather similar to Karyotaki et al. (30) in that roughly half of clients showed improvement following ICBT and remission was achieved by a third.

Given these estimates, the outstanding question is how well this compares against face-to-face CBT. As previously mentioned, there are a few studies in which clients have been randomly assigned either face-to-face CBT or therapist-guided CBT. In the most recent of such study, Carlbring et al. (5) found no differences in effect. The response rates in CBT across different disorders and conditions are difficult to estimate; as to our knowledge, there is no similar IPDMA on response rates in face-to-face CBT. The closest we can get is a meta-analysis on anxiety disorders based on published data (28). In that review, 31% of the studies had defined response using RCI, and the results were similar to the range found in this analysis (44.5–51.1%). However, Loerinc et al. (28) also reported that the use of RCI in combination with a clinical cut-off (as was done in the present study) was associated with a 28% lower response rate (or, as expressed in this study, the remission rate was 28% lower than the RCI response rate). The difference we found was somewhat larger, as about half of the responders also showed remission. From a more naturalistic perspective, Gyani et al. (82) reported that 63.7% of participants in their clinical sample showed reliable improvement following evidence-based face-to-face treatment. In a recent meta-analysis, Springer et al. (83) reported a remission estimate as high as 51% (compared to our estimate of 35%). As stated by Loerinc et al. (28), there is a large variation in how to define both response and remission in CBT trials; thus, one advantage of the approach taken here is that we can use the same approach across studies. However, the definition of remission used (two SDs below the pre-treatment mean) was impossible in some studies (leading us to correct that estimate) and unrealistic in others. Another disadvantage of a statistical definition of response and remission is that it is heavily dependent on the sample upon which it is calculated. This pertains to both RCI and recovery as outlined by Jacobson and Truax (29). Another approach would have been to determine criteria for response and remission independently of the study, which is possible when there is data on non-clinical samples. For example, on the Beck Depression Inventory (44), a 10-point reduction could be seen as indicative of response, and 13-point reduction indicative of recovery. Applying these criteria for the Stella trial (see **Table 3**; 63), in which 57.5% of participants responded according to the RCI and 0% remitted, the corresponding figures of using a 10-point reduction (53.1%) and a score of 13 or below on the BDI (59.4%) paints a slightly different picture (although the results are similar between the RCI criteria and the 10-point reduction). In particular, the difference between the 0% classified as remitters *versus* the 59.4% having a score indicating minimal depression (44) shows the importance of response definitions (for a detailed discussion, see 37). However, in the present IPDMA, we found it to be of value to use similar criteria across trials. Future research could focus more on the external criteria of improvement instead of study-specific criteria. Unfortunately, the BDI is an exception, being widely used in many trials, and for some conditions and measures included in this IPDMA, cut-off scores and non-clinical norms have not been established.

In the present IPDMA, we conducted exploratory analyses to see if response could be predicted. It is important to note that there was no firm theoretical basis for our selection of predictors and, therefore, our findings must be interpreted with caution, as the identification of a significant predictor could have practical implications for future treatment recommendations. This is particularly the case when findings are based on samples larger than is commonly used in psychotherapy trials. However, the finding that symptom severity was not a negative predictor, but rather the opposite is in line with a previous IPDMA on low-intensity interventions for depression (33). From a clinical point of view, this makes sense, as having symptoms makes treatment more relevant than if subclinical or even different symptoms are present in the client. However, it also suggests that our ICBT trials probably included participants without severe symptoms. It is therefore possible that this finding is based on selection criteria used in trials and not necessarily relevant for clinical practice when treatment is offered with fewer restrictions. In contrast to the finding for response, we found a small negative effect of pre-treatment severity for the prediction of remission. These two conflicting results may indicate that large improvements are less likely if the client has more symptoms. However, as remission requires a low level of symptoms, it is less likely that a client with many symptoms will reach that low level. As in treatment research in general, it could be that the search for predictors is best pursued in ordinary clinical settings rather than in well-controlled trials. On a promising note, large ICBT effectiveness studies are being reported (84), which may provide clearer insight into the efficacy of different approaches for different populations.

The odds ratio in favor of female participants was surprisingly high (OR = 2.22), which is hard to explain as this is not a consistent result from previous individual trials. One possible explanation is the fact that the gender proportions in trials are nested with the condition treated. For example, in a typical depression trial, there is a majority of women, whereas in other conditions, there are more or less equal proportions of men and women, while some studies have only men (e.g., erectile dysfunction). The overall takeaway message here is that this finding needs further investigation in future trials to confirm its veracity. Gender was not found to be predictive of remission.

Interestingly, while the IPDMA by Karyotaki et al. (30) found old age to be weakly associated with better response (OR = 1.01), there was no such effect in this study suggesting that age is not a predictor of outcome. In Karyotaki et al.’s (30) complete case analyses, baseline severity (OR = 1.16) was found to predict better outcome, which was in line with our findings (but not in their intention to treat analyses). Gender did not predict outcome in Karyotaki et al.’s (30) IPDMA, but, again, their review focused on only depression which means a larger portion of the population was likely female.

In this IPDMA, we found decreased odds for responding to treatment when having an anxiety disorder as compared to depression/mood disorders or other (erectile dysfunction, relationship problems, and gambling disorder). This was a small effect, but still puzzling and hard to understand given that the overall picture is the opposite—indicating a higher response to ICBT in clients with anxiety disorders. Anxiety disorders were also found to be predictive of lower rates of remission.

While non-significant findings cannot be viewed as proof of absence of an effect, it is still interesting that ICBT has had marginal success in finding predictors of change as well as moderators and mediators of outcome (1). There are several possible reasons for this. First, participants in trials are not selected for their differences. Rather, they are chosen based on diagnostic criteria, access to the Internet, willingness to be a research participant, etc., all of which likely reduce the chance of identifying accurate predictors. Second, predictors of dropout (85) may ultimately be more illuminating, even if a given IPDMA was on self-guided ICBT for depressive symptoms. It is, however, interesting to note that they found that male gender (RR = 1.08) and co-morbid anxiety symptoms (RR = 1.18) significantly increased the risk of dropping out of the study, which is in line with our findings with regards to gender and anxiety disorders. In spite of the overall inconsistency in findings and lack of findings, we believe that IPDMA is a powerful and reliable tool for answering questions regarding predictors of response (86). However, this will require concerted efforts to align both outcome measures as well as data on predictors in order to make studies comparable. Even if we were co-workers and principal investigators in the trials included in this IPDMA, the data were not consistent with regards to background variables etc. It becomes even more problematic when attempting to combine datasets from different research groups.

This study had several limitations. We focus on four, knowing that there are further objections that could be raised. First, we only included our own trials, which were conducted in Sweden. IPDMAs are often selective, as original data sometimes cannot be obtained from authors, but in this case, we cannot generalize the results outside of our own culture and setting. In addition to not including trials from international groups, we also did not include the most recent and unpublished trials from our own groups, and there are other groups in Sweden with trials that were also not included. Furthermore, with the focus on our own studies conducted over a period close to 20 years, there is a possibility of time trends, which we did not focus on in this study. Technological changes may not influence effects, but, to give an example, early studies relied more on printing out text materials (87), whereas recent studies are typically delivered *via* online platforms (e.g., responsive to the presentation format, such as smartphones or computers) (2). Second, even if this limitation is not unique for ICBT, outcome measures were largely based on self-report. Such measures are useful and generally have good psychometric properties but still present a possible risk that self-reported changes do not correspond with actual behavior changes and do not conform to the findings of an interview. Third, as commented on by Loerinc et al. (28), treatment response can be defined by several measures, but here, we focused only on the primary outcomes. Our dataset on IPDMA could also be used to analyze effects on other secondary measures of constructs, like quality of life, as several of our studies have used the same measure for this construct (e.g., 88), and it has been reported that the effects of ICBT might be lower on that construct (89). The fourth limitation relates to the statistical methods used. We decided to report on a complete case basis, but we could not exclude bias, as missing data was not considered.

## Conclusions

In spite of the limitations, the present IPDMA suggests that ICBT can lead to major reductions in symptoms and that more than half of clients, on average, respond to treatment. A lower proportion remits; thus, there is room for improvement. It is possible that women benefit more from ICBT based on our findings, and symptom severity seems to be predictive of outcome, but these findings should be interpreted with caution. Our findings that studies on clients with anxiety disorders were associated with less response is also notable but should be regarded with care. Future IPDMAs should include more trials and should also consider secondary outcomes, such as quality of life.

## Data Availability Statement

The datasets analyzed in this manuscript are not publicly available. Requests to access the datasets should be directed to gerhard.andersson@liu.se.

## Author Contributions

All of the authors contributed in the process of completing the current study and writing the final manuscript. AR conducted the aggregation of raw scores into a single data matrix and completed the statistical analysis with input from GA and PC. GA drafted the first version of the text and did additional calculations, while AR and PC provided feedback and reviewed and revised the manuscript.

## Conflict of Interest

The authors declare that the research was conducted in the absence of any commercial or financial relationships that could be construed as a potential conflict of interest.

## References

[1] Andersson G Internet interventions: past, present and future. Internet Interv (2018) 12:181–8. 10.1016/j.invent.2018.03.008 PMC6096319 30135782

[2] Vlaescu G Alasjö A Miloff A Carlbring P Andersson G Features and functionality of the Iterapi platform for internet-based psychological treatment. Internet Interv (2016) 6:107–14. 10.1016/j.invent.2016.09.006 PMC6096305 30135819

[3] Andersson G The internet and CBT: a clinical guide. Boca Raton: CRC Press (2015). 10.1201/b13645

[4] Baumeister H Reichler L Munzinger M Lin J The impact of guidance on Internet-based mental health interventions—a systematic review. Internet Interv (2014) 1:205–15. 10.1016/j.invent.2014.08.003

[5] Carlbring P Andersson G Cuijpers P Riper H Hedman-Lagerlöf E Internet-based vs. face-to-face cognitive behavior therapy for psychiatric and somatic disorders: an updated systematic review and meta-analysis. Cogn Behav Ther (2018) 47:1–18. 10.1080/16506073.2017.1401115 29215315

[6] Andersson G Rozental A Shafran R Carlbring P Long-term effects of internet-supported cognitive behavior therapy. Expert Rev Neurother (2018) 18:21–8. 10.1080/14737175.2018.1400381 29094622

[7] Andersson G Hedman E Effectiveness of guided Internet-delivered cognitive behaviour therapy in regular clinical settings. Verhaltenstherapie (2013) 23:140–8. 10.1159/000354779

[8] Vigerland S Lenhard F Bonnert M Lalouni M Hedman E Ahlen J Internet-delivered cognitive behavior therapy for children and adolescents: a systematic review and meta-analysis. Clin Psychol Rev (2016) 50:1–10. 10.1016/j.cpr.2016.09.005 27668988

[9] Andersson G Carlbring P Titov N Lindefors N (2019). Internet interventions for adults with anxiety and mood disorders: A narrative umbrella review of recent meta-analyses. Can J Psychiatry 64: 465–470. 10.1177/0706743719839381 31096757 PMC6610559

[10] Dear BF Zou JB Ali S Lorian CN Johnston L Sheehan J Clinical and cost-effectiveness of therapist-guided internet-delivered cognitive behavior therapy for older adults with symptoms of anxiety: a randomized controlled trial. Behav Ther (2015b) 46:206–17. 10.1016/j.beth.2014.09.007 25645169

[11] Nygren T Brohede D Koshnawa K Osmana SS Johansson R Andersson G Internet-based treatment of depressive symptoms in a Kurdish population: a randomized controlled trial. J Clin Psychol (2019) 75:985–98. 10.1002/jclp.22753 30702758

[12] Rozental A Forsell E Svensson A Andersson G Carlbring P Internet-based cognitive behavior therapy for procrastination: a randomized controlled trial. J Consult Clin Psychol (2015) 83:808–24. 10.1037/ccp0000023 25939016

[13] Sijbrandij M Kunovski I Cuijpers P Effectiveness of internet-delivered cognitive behavioral therapy for posttraumatic stress disorder: a systematic review and meta-analysis. Depress Anxiety (2016) 33:783–91. 10.1002/da.22533 27322710

[14] Păsărelu C Andersson G Bergman Nordgren L Dobrean A Internet-delivered transdiagnostic and tailored cognitive behavioral therapy for anxiety and depression: a systematic review and meta-analysis. Cogn Behav Ther (2017) 46:1–28. 10.1080/16506073.2016.1231219 27712544

[15] Dear BF Staples LG Terides MD Karin E Zou J Johnston L Transdiagnostic versus disorder-specific and clinician-guided versus self-guided internet-delivered treatment for generalized anxiety disorder and comorbid disorders: a randomized controlled trial. J Anxiety Disord (2015a) 36:63–77. 10.1016/j.janxdis.2015.09.003 26460536

[16] Johansson R Hesslow T Ljotsson B Jansson A Jonsson L Färdig S Internet-based affect-focused psychodynamic therapy for social anxiety disorder: a randomized controlled trial with 2-year follow-up. Psychotherapy (2017) 54:351–60. 10.1037/pst0000147 29251954

[17] Donker T Bennett K Bennett A Mackinnon A van Straten A Cuijpers P Internet-delivered interpersonal psychotherapy versus internet-delivered cognitive behavioral therapy for adults with depressive symptoms: randomized controlled noninferiority trial. J Med Internet Res (2013) 15:e82. 10.2196/jmir.2307 23669884 PMC3668608

[18] Dahlin M Andersson G Magnusson K Johansson T Sjögren J Håkansson A Internet-delivered acceptance-based behaviour therapy for generalized anxiety disorder: a randomized controlled trial. Behav Res Ther (2016) 77:86–95. 10.1016/j.brat.2015.12.007 26731173

[19] Boettcher J Berger T Renneberg B Internet-based attention training for social anxiety: a randomized controlled trial. Cogn Ther Res (2012) 36:552–36. 10.1007/s10608-011-9374-y

[20] *Ström M Uckelstam C-J Andersson G Hassmén P Umefjord G Carlbring P Internet-delivered therapist-guided physical activity for mild to moderate depression: a randomized controlled trial. PeerJ (2013) 1:e178. 10.7717/peerj.178 24109561 PMC3792189

[21] *Boettcher J Åstrom V Pahlsson D Schenstrom O Andersson G Carlbring P Internet-based mindfulness treatment for anxiety disorders: a randomized controlled trial. Behav Ther (2014) 45:241–53. 10.1016/j.beth.2013.11.003 24491199

[22] Carlbring P Ekselius L Andersson G Treatment of panic disorder *via *the Internet: a randomized trial of CBT vs. applied relaxation. J Behav Ther Exp Psychiatry (2003) 34:129–40. 10.1016/S0005-7916(03)00026-0 12899896

[23] *Paxling B Almlöv J Dahlin M Carlbring P Breitholtz E Eriksson T Guided internet-delivered cognitive behavior therapy for generalized anxiety disorder: a randomized controlled trial. Cogn Behav Ther (2011) 40:159–73. 10.1080/16506073.2011.576699 21770848

[24] Hedman E Andersson E Andersson G Lindefors N Lekander M Rück C Mediators in Internet-based cognitive behavior therapy for severe health anxiety. PLoS One (2013) 8:e77752. 10.1371/journal.pone.0077752 24147073 PMC3798404

[25] Bendelin N Hesser H Dahl J Carlbring P Zetterqvist Nelson K Andersson G Experiences of guided Internet-based cognitive-behavioural treatment for depression: a qualitative study. BMC Psychiatry (2011) 11:107. 10.1186/1471-244X-11-107 21718523 PMC3142491

[26] Rozental A Magnusson K Boettcher J Andersson G Carlbring P For better or worse: an individual patient data meta-analysis of deterioration among participants receiving Internet-based cognitive behavior therapy. J Consult Clin Psychol (2017) 85:160–77. 10.1037/ccp0000158 27775414

[27] Rozental A Andersson G Carlbring P In the absence of effects: an individual patient data meta-analysis of non-response and its predictors in internet-based cognitive behavior therapy. Front Psychol (2019) 10:589. 10.3389/fpsyg.2019.00589 30984061 PMC6450428

[28] Loerinc AG Meuret AE Twohig MP Rosenfield D Bluett EJ Craske MG Response rates for CBT for anxiety disorders: need for standardized criteria. Clin Psychol Rev (2015) 42:72–82. 10.1016/j.cpr.2015.08.004 26319194

[29] Jacobson NS Truax P Clinical significance: a statistical approach to defining meaningful change in psychotherapy research. J Consult Clin Psychol (1991) 59:12–9. 10.1037/0022-006X.59.1.12 2002127

[30] Karyotaki E Ebert DD Donkin L Riper H Twisk J Burger S Do guided Internet-based interventions result in clinically relevant changes for patients with depression? An individual participant data meta-analysis. Clin Psychol Rev (2018) 63:80–92. 10.1016/j.cpr.2018.06.007 29940401

[31] Stewart L Tierney JF To IPD or not to IPD? Advantages and disadvantages of systematic reviews using individual patient data. Eval Health Prof (2002) 25(1):76–97. 10.1177/0163278702025001006 11868447

[32] Simmonds MC Higgins JPT Stewart LA Tierney JF Clarke MJ Thompson SG Meta-analysis of individual patient data from randomized trials: a review of methods used in practice. Clin Trials (2005) 2(3):209–17. 10.1191/1740774505cn087oa 16279144

[33] Bower P Kontopantelis E Sutton AP Kendrick T Richards D Gilbody S Influence of initial severity of depression on effectiveness of low intensity interventions: meta-analysis of individual patient data. Br Med J (2013) 346:f540. 10.1136/bmj.f540 23444423 PMC3582703

[34] Riper H Hoogendoorn A Cuijpers P Karyotaki E Boumparis N Pastor AM Effectiveness and treatment moderators of internet interventions for adult problem drinking: an individual patient data meta-analysis of 19 randomised controlled trials. Plos Med (2018) 15:e1002714. 10.1371/journal.pmed.1002714 30562347 PMC6298657

[35] Mukaka M White SA Terlouw DJ Mwapasa V Kalilani-Phiri L Faragher EB Is using multiple imputation better than complete case analysis for estimating a prevalence (risk) difference in randomized controlled trials when binary outcome observations are missing? Trials (2016) 17:341. 10.1186/s13063-016-1473-3 27450066 PMC4957845

[36] Akl EA Kahale LA Agoritsas T Brignardello-Petersen R Busse JW Carrasco-Labra A Handling trial participants with missing outcome data when conducting a meta-analysis: a systematic survey of proposed approaches. Syst Rev (2015) 4:98–8. 10.1186/s13643-015-0083-6 PMC4511978 26202162

[37] Jacobson NS Roberts LJ Berns SB McGlinchey JB Methods for defining and determining the clinical significance of treatment effects: description, application, and alternatives. J Consult Clin Psychol (1999) 67:300–7. 10.1037/0022-006X.67.3.300 10369050

[38] Evans C Margison F Barkham M The contribution of reliable and clinically significant change methods to evidence-based mental health. Evid-Based Ment Health (1998) 1(3):70–2. 10.1136/ebmh.1.3.70

[39] Saemundsson BR Borsdottir F Kristjansdottir H Olason DB Smari J Sigurdsson JF Psychometric properties of the Icelandic version of the Beck Anxiety Inventory in a clinical and a student population. Eur J Psychol Assess (2011) 27:133–41. 10.1027/1015-5759/a000059

[40] Heeren A Maurange P Rossignol M Vanhaelen M Peschard V Eeckhout C Self-report version of the Liebowitz Social Anxiety Scale: psychometric properties of the French version. Can J Behav Sci (2012) 44:99–107. 10.1037/a0026249

[41] Lee EH Kim JH Yu BH Reliability and validity of the self-report version of the Panic Disorder Severity Scale in Korea. Depress Anxiety (2009) 26:E120–3. 10.1002/da.20461 19373866

[42] Zuithoff NPA Vergouwe Y Kling M Nazareth I van Wezep MJ Moons KGM The Patient Health Questionnaire-9 for detection of major depressive disorder in primary care: consequences of current thresholds in a crossectional study. BMC Fam Pract (2010) 11:98. 10.1186/1471-2296-11-98 21144018 PMC3009622

[43] Rosen RC Riley A Wagner G Osterloh IH Kirkpatrick J Mishra A The international index of erectile function (IIEF): a multidimensional scale for assessment of erectile dysfunction. Urology (1997) 49:822–30. 10.1016/S0090-4295(97)00238-0 9187685

[44] Beck AT Steer RA Beck depression inventory. Manual, Svensk version (Swedish version). Fagernes: Psykologiförlaget AB (1996). 10.1037/t00742-000

[45] Sundin EC Horowitz MJ Impact of Event Scale: psychometric properties. Br J Psychiatry (2002) 180:205–9. 10.1192/bjp.180.3.205 11872511

[46] Spitzer RL Kroenke K Williams JB Löwe B A brief measure for assessing generalized anxiety disorder: the GAD-7. Arch Intern Med (2006) 166:1092–7. 10.1001/archinte.166.10.1092 16717171

[47] Pallesen S Nordhus IH Carlstedt B Thayer JF Johnsen TB A norwegian adaption of the penn state worry questionnaire: factor structure, reliability, validity and norms. Scand J Psychol (2006) 47:281–91. 10.1111/j.1467-9450.2006.00518.x 16869861

[48] Arrindell WA The fear of fear concept: stability, retest artifact and predictive power. Behav Res Ther (1993) 31:139–48. 10.1016/0005-7967(93)90065-3 8442739

[49] Carey MP Spector IP Lantinga LJ Krauss DJ Reliability of the Dyadic Adjustment Scale. Psychol Assess (1993) 5:238–40. 10.1037/1040-3590.5.2.238

[50] Klorman R Weerts TC Hastings JE Melamed BG Lang PJ Psychometric properties of some specific-fear questionnaires. Behav Ther (1974) 5:401–9. 10.1016/S0005-7894(74)80008-0

[51] Fantino B Moore N The self-reported Montgomery-Åsberg Depression rating scale is a useful evaluative tool in major depressive disorder. BMC Psychiatry (2009) 9:26. 10.1186/1471-244X-9-26 19473506 PMC2701427

[52] Muris P Merckelbach H A comparison of two spider fear questionnaires. J Behav Ther Exp Psychiatry (1996) 27:241–4. 10.1016/S0005-7916(96)00022-5 8959425

[53] Gerstein DR Volberg RA Toce MT Harwood H Johnson RA Buie T Gambling Impact and Behavior Study: Report to the National Gambling Impact Study Commission. Chicago, IL: NORC, at the University of Chicago (1999).

[54] Hedman E Andersson E Ljótsson B Andersson G Andersson EM Schalling M Clinical and genetic outcome determinants of Internet- and group-based cognitive behavior therapy for social anxiety disorder. Acta Psychiatr Scand (2012) 126:126–36. 10.1111/j.1600-0447.2012.01834.x 22320999

[55] jamovi project. jamovi (Version 0.9) [Computer Software] (2018). Retrieved from http://www.jamovi.org.

[56] First MB Gibbon M Spitzer RL Williams JB Structured clinical interview for DSM-IV Axis I Disorders (SCID-I). Washington, D.C: American Psychiatric Press (1997).

[57] Sheehan DV Lecrubier Y Sheehan KH Amorim P Janavs J Weiller E The Mini-International Neuropsychiatric Interview (MINI): the development and validation of a structured diagnostic psychiatric interview for DSM-IV and ICD-10. J Clin Psychiatry (1998) 59:22–33. 10.1037/t18597-000 9881538

[58] Blake DD Weathers FW Nagy LM Kaloupek DG Gusman FD Charney DS The development of a clinician-administered PTSD scale. J Trauma Stress (1995) 8(1):75–90. 7712061 10.1007/BF02105408

[59] *Ivanova E Lindner P Ly KH Dahlin M Vernmark K Andersson G Guided and unguided Acceptance and Commitment Therapy for social anxiety disorder and/or panic disorder provided *via *the Internet and a smartphone application: a randomized controlled trial. J Anxiety Disord (2016) 44:27–35. 10.1016/j.janxdis.2016.09.012 27721123

[60] Nyström MBT Stenling A Sjöström E Neely G Lindner P Hassmén P Behavioral activation versus physical activity *via the* Internet: a randomized controlled trial. J Affect Disord (2017) 215:85–93. 10.1016/j.jad.2017.03.018 28319696

[61] *Andersson E Walen C Hallberg J Paxling B Dahlin M Almlov J A randomized controlled trial of guided Internet-delivered cognitive behavioral therapy for erectile dysfunction. J Sex Med (2011) 8:2800–9. 10.1111/j.1743-6109.2011.02391.x 21797983

[62] *Boettcher J Magnusson K Marklund A Berglund E Blomdahl R Braun U Adding a smartphone app to Internet-based self-help for social anxiety: a randomized controlled trial. Comput Hum Behav (2018) 87:98–108. 10.1016/j.chb.2018.04.052

[63] Andersson G Hesser H Veilord A Svedling L Andersson F Sleman O Randomised controlled non-inferiority trial with 3-year follow-up of internet-delivered versus face-to-face group cognitive behavioural therapy for depression. J Affect Disord (2013a) 151:986–94. 10.1016/j.jad.2013.08.022 24035673

[64] *Carlbring P Hägglund M Luthström A Dahlin M Kadowaki Å Vernmark K Internet-based behavioral activation and acceptance-based treatment for depression: a randomized controlled trial. J Affect Disord (2013) 148:331–7. 10.1016/j.jad.2012.12.020 23357657

[65] *Ivarsson D Blom M Hesser H Carlbring P Enderby P Nordberg R Guided Internet-delivered cognitive behaviour therapy for post-traumatic stress disorder: a randomized controlled trial. Internet Interv (2014) 1:33–40. 10.1016/j.invent.2014.03.002

[66] *Vernmark K Lenndin J Bjärehed J Carlsson M Karlsson J Öberg J Internet administered guided self-help versus individualized e-mail therapy: a randomized trial of two versions of CBT for major depression. Behav Res Ther (2010) 48(5):368–76. 10.1016/j.brat.2010.01.005 20152960

[67] *Carlbring P Maurin L Törngren C Linna E Eriksson T Sparthan E Individually-tailored, Internet-based treatment for anxiety disorders: a randomized controlled trial. Behav Res Ther (2011) 49(1):18–24. 10.1016/j.brat.2010.10.002 21047620

[68] *Nordgren LB Hedman E Etienne J Bodin J Kadowaki A Eriksson S Effectiveness and cost-effectiveness of individually tailored Internet-delivered cognitive behavior therapy for anxiety disorders in a primary care population: a randomized controlled trial. Behav Res Ther (2014) 59:1–11. 10.1016/j.brat.2014.05.007 24933451

[69] *Andersson G Paxling B Roch-Norlund P Östman G Norgren A Almlöv J Internet-based psychodynamic versus cognitive behavioral guided self-help for generalized anxiety disorder: a randomized controlled trial. Psychother Psychosom (2012) 81:344–55. 10.1159/000339371 22964540

[70] *Carlbring P Nilsson-Ihrfelt E Waara J Kollenstam C Buhrman M Kaldo V Treatment of panic disorder: live therapy vs. self-help *via *the Internet. Behav Res Ther (2005) 43(10):1321–33. 10.1016/j.brat.2004.10.002 16086983

[71] Andersson G Waara J Jonsson U Malmaeus F Carlbring P Öst L-G Internet-Based exposure treatment versus one-session exposure treatment of snake phobia: A randomized controlled trial. Cogn Behav Ther (2013b) 42:284–91. 10.1080/16506073.2013.844202 24245707

[72] *Boettcher J Hasselrot J Sund E Andersson G Carlbring P Combining attention training with Internet-based cognitive-behavioural self-help for social anxiety: a randomised controlled trial. Cogn Behav Ther (2013) 43:34–48. 10.1080/16506073.2013.809141 23898817 PMC3869050

[73] *Kuckertz JM Gildebrant E Liliequist B Karlstrom P Vappling C Bodlund O Moderation and mediation of the effect of attention training in social anxiety disorder. Behav Res Ther (2014) 53:30–40. 10.1016/j.brat.2013.12.003 24373984

[74] *Dagöö J Asplund RP Bsenko HA Hjerling S Holmberg A Westh S Cognitive behavior therapy versus interpersonal psychotherapy for social anxiety disorder delivered *via* smartphone and computer: a randomized controlled trial. J Anxiety Disord (2014) 28:410–7. 10.1016/j.janxdis.2014.02.003 24731441

[75] *Andersson G Carlbring P Holmstrom A Sparthan E Furmark T Nilsson-Ihrfelt E Internet-based self-help with therapist feedback and *in vivo* group exposure for social phobia: a randomized controlled trial. J Consult Clin Psychol (2006) 74(4):677–86. 10.1037/0022-006X.74.4.677 16881775

[76] *Carlbring P Gunnarsdóttir M Hedensjö L Andersson G Ekselius L Furmark T Treatment of social phobia from a distance: a randomized trial of internet delivered cognitive behavior therapy (CBT) and telephone support. Br J Psychiatry (2007) 190:123–8. 10.1192/bjp.bp.105.020107 17267928

[77] *Tillfors M Carlbring P Furmark T Lewenhaupt S Spak M Eriksson A Treating university students with social phobia and public speaking fears: Internet delivered self-help with or without live group exposure sessions. Depress Anxiety (2008) 25:708–17. 10.1002/da.20416 18729147

[78] *Furmark T Carlbring P Hedman E Sonnenstein A Clevberger P Bohman B Bibliotherapy and Internet delivered cognitive behaviour therapy with additional guidance for social anxiety disorder: randomised controlled trial. Br J Psychiatry (2009) 195:440–7. 10.1192/bjp.bp.108.060996 19880935

[79] *Silfvernagel K Westlinder A Andersson S Bergman K Hernandez RD Fallhagen L Individually tailored internet-based cognitive behaviour therapy for older adults with anxiety and depression: a randomised controlled trial. Cogn Behav Ther (2018) 47:286–300. 10.1080/16506073.2017.1388276 29068266

[80] *Andersson G Waara J Jonsson U Malmaeus F Carlbring P Öst LG Internet-based self-help versus one-session exposure in the treatment of spider phobia: a randomized controlled trial. Cogn Behav Ther (2009) 38:114–20. 10.1080/16506070902931326 20183690

[81] *Carlbring P Degerman N Jonsson J Andersson G Internet-based treatment of pathological gambling with a three-year follow-up. Cogn Behav Ther (2012) 41:321–34. 10.1080/16506073.2012.689323 PMC3516818 22620990

[82] Gyani A Shafran R Layard R Clark DM Enhancing recovery rates: lessons from year one of IAPT. Behav Res Ther (2013) 51:597–606. 10.1016/j.brat.2013.06.004 23872702 PMC3776229

[83] Springer KS Levy HC Tolin DF Remission in CBT for adult anxiety disorders: a meta-analysis. Clin Psychol Rev (2018) 61:1–8. 10.1016/j.cpr.2018.03.002 29576326

[84] Titov N Dear BF Staples L Bennett-Levy J Klein B Rapee RM The first 30 months of the MindSpot Clinic: Evaluation of a national e-mental health service against project objectives. Aust N Z J Psychiatry (2017) 51:1227–39. 10.1177/0004867416671598 27733709

[85] Karyotaki E Kleiboer A Smit F Turner DT Mira Pastor A Andersson G Predictors of treatment dropout in self-guided web-based interventions for depression: an ‘‘individual patient data’’ meta-analysis. Psychol Med (2015) 45:2717–26. 10.1017/S0033291715000665 25881626

[86] Oxman AD Clarke MJ Stewart LA From science to practice: meta-analyses using individual patient data are needed. JAMA (1995) 274(10):845–6. 10.1001/jama.1995.03530100085040 7650811

[87] Andersson G Bergström J Buhrman M Carlbring P Holländare F Kaldo V Development of a new approach to guided self-help *via *the Internet. Swedish Exp J Technol Hum Serv (2008) 26:161–81. 10.1080/15228830802094627

[88] Frisch MB Cornell J Villanueva M Retzlaff PJ Clinical validation of the quality of life inventory: a measure of life satisfaction for use in treatment planning and outcome assessment. Psychol Assess (1992) 4:92–101. 10.1037/1040-3590.4.1.92

[89] Hofmann SG Wu JQ Boettcher H Effect of cognitive-behavioral therapy for anxiety disorders on quality of life: a meta-analysis. J Consult Clin Psychol (2014) 82:375–91. 10.1037/a0035491 PMC4029924 24447006

