# Supplementary material for: Response and Remission Rates in Internet-Based Cognitive Behavior Therapy: An Individual Patient Data Meta-Analysis
Source: Front Psychiatry. 2019 Oct 25;10:749. doi: 10.3389/fpsyt.2019.00749 (PMC6823683; doi:10.3389/fpsyt.2019.00749)
Supplement: Supplementary file 1 [file DataSheet_1.pdf]

```
jmv::logRegBin(
   data = data,
   dep = IMPROVE_TOTAL,
   covs = vars(ZTOTAL, AGE),
   factors = vars(CIVILSTATUS, PRIOR_PSYCH, PRIOR_MEDS, SICK_LEAVE,
EDUCATION_PREDICTOR_DUMMY, DIAGNOSIS_PREDICTOR_DUMMY, GENDER),
   blocks = list(
      list(
         "ZTOTAL",
         "CIVILSTATUS",
         "PRIOR_PSYCH",
         "PRIOR_MEDS",
         "SICK_LEAVE",
         "EDUCATION_PREDICTOR_DUMMY",
         "AGE",
         "DIAGNOSIS_PREDICTOR_DUMMY",
         "GENDER")),
   refLevels = list(
      list(
         var="IMPROVE_TOTAL",
         ref="No"),
      list(
         var="CIVILSTATUS",
         ref="Single"),
      list(
         var="PRIOR_PSYCH",
         ref="No"),
      list(
         var="PRIOR_MEDS",
         ref="No"),
      list(
         var="SICK_LEAVE",
         ref="No"),
      list(
         var="EDUCATION_PREDICTOR_DUMMY",
         ref="Below university level"),
      list(
         var="DIAGNOSIS_PREDICTOR_DUMMY",
         ref="Depression and other"),
      list(
         var="GENDER",
         ref="Male")),
   modelTest = TRUE,
   bic = TRUE,
   pseudoR2 = NULL,
   OR = TRUE,
   ciOR = TRUE,
```

```
    collin = TRUE)

jmv::logRegBin(
    data = data,
    dep = RECOVERY_ADJUSTFLOOREFFECT_TOTAL,
    covs = vars(ZTOTAL, AGE),
    factors = vars(CIVILSTATUS, PRIOR_PSYCH, PRIOR_MEDS, SICK_LEAVE,
EDUCATION_PREDICTOR_DUMMY, DIAGNOSIS_PREDICTOR_DUMMY, GENDER),
    blocks = list(
        list(
            "ZTOTAL",
            "CIVILSTATUS",
            "PRIOR_PSYCH",
            "PRIOR_MEDS",
            "SICK_LEAVE",
            "EDUCATION_PREDICTOR_DUMMY",
            "AGE",
            "DIAGNOSIS_PREDICTOR_DUMMY",
            "GENDER")),
    refLevels = list(
        list(
            var="RECOVERY_ADJUSTFLOOREFFECT_TOTAL",
            ref="No"),
        list(
            var="CIVILSTATUS",
            ref="Single"),
        list(
            var="PRIOR_PSYCH",
            ref="No"),
        list(
            var="PRIOR_MEDS",
            ref="No"),
        list(
            var="SICK_LEAVE",
            ref="No"),
        list(
            var="EDUCATION_PREDICTOR_DUMMY",
            ref="Below university level"),
        list(
            var="DIAGNOSIS_PREDICTOR_DUMMY",
            ref="Depression and other"),
        list(
            var="GENDER",
            ref="Male")),
    modelTest = TRUE,
    bic = TRUE,
    pseudoR2 = NULL,
```

```
    OR = TRUE,
    ciOR = TRUE,
    collin = TRUE)

jmv::logRegBin(
    data = data,
    dep = IMPROVE_TOTAL,
    covs = PUBLICATIONYEAR,
    blocks = list(
        list(
            "PUBLICATIONYEAR")),
    refLevels = list(
        list(
            var="IMPROVE_TOTAL",
            ref="No")),
    modelTest = TRUE,
    bic = TRUE,
    pseudoR2 = NULL,
    OR = TRUE,
    ciOR = TRUE,
    collin = TRUE)
```
